# Toxicity Assessment of (4*Z*)-Lachnophyllum and (4*Z*,8*Z*)-Matricaria Lactones: Implications for Environmental Safety of Bioherbicides

**DOI:** 10.3390/toxins17040169

**Published:** 2025-04-01

**Authors:** Edith Guadalupe Padilla Suarez, Jesús G. Zorrilla, Marisa Spampinato, Teresa Pannullo, Francesca Esposito, Mónica Fernández-Aparicio, Giovanni Libralato, Antonietta Siciliano, Marco Masi, Alessio Cimmino

**Affiliations:** 1 Department of Biology, University of Naples Federico II, Complesso Universitario Monte Sant’Angelo, Via Cintia 4, 80126 Naples, Italy; edith.padilla@unina.it (E.G.P.S.); marisa.spampinato@unina.it (M.S.); teresa_pannullo@virgilio.it (T.P.); francy-esposito2011@libero.it (F.E.); giovanni.libralato@unina.it (G.L.); 2 Department of Chemical Sciences, University of Naples Federico II, Complesso Universitario Monte Sant’ Angelo, Via Cintia, 80126 Naples, Italy; jesus.zorrilla@uca.es (J.G.Z.); alessio.cimmino@unina.it (A.C.); 3 Allelopathy Group, Department of Organic Chemistry, Facultad de Ciencias, Institute of Biomolecules (INBIO), University of Cadiz, C/Avenida República Saharaui, s/n, 11510 Puerto Real, Spain; 4 Department of Crop Protection, Institute for Sustainable Agriculture (IAS), CSIC, Avenida Menéndez Pidal s/n, 14004 Córdoba, Spain; monica.fernandez@ias.csic.es

**Keywords:** ecotoxicology, natural products, broomrape, acetylenic furanones, bioherbicides, parasitic weed management

## Abstract

(4*Z*,8*Z*)-Matricaria lactone (MAT) and (4*Z*)-lachnophyllum lactone (LAC) are natural acetylenic furanones with bioherbicidal potential. This study evaluates their possibilities and ecotoxicological impact on aquatic (*Aliivibrio fischeri*, *Raphidocelis subcapitata*, and *Daphnia magna*) and terrestrial (*Caenorhabditis elegans*, *Lepidum sativum*) model organisms. MAT exhibited rapid degradation, with 90% decomposition within 24 h and over 98% by day 16, while LAC was more stable, degrading by only 8.5% in 24 h and 67% by day 16. Despite its rapid breakdown, MAT exhibited higher acute toxicity to *A. fischeri* (EC_10_ = 0.063 mg L^−1^; EC_50_ = 0.642 mg L^−1^) compared to LAC (EC_10_ = 0.524 mg L^−1^; EC_50_ = 8.078 mg L^−1^). Toxicity patterns in *R. subcapitata* differed, with MAT promoting slightly higher growth compared to the control, suggesting hormetic effects (EC_10_ = 3.417 mg L^−1^; EC_50_ = 4.520 mg L^−1^), while LAC inhibited growth concentration (EC_10_ = 0.304 mg L^−1^; EC_50_ = 9.880 mg L^−1^). Both compounds immobilized *D. magna*, with LAC showing greater delayed toxicity (EC_50_ = 1.728 mg L^−1^ vs. MAT EC_50_ = 2.239 mg L^−1^). Furthermore, for *L. sativum*, there were no effects on the germination, but effects were observed in the lengths of the shoots (LAC EC_50_ = 85.89 mg L^−1^ vs. MAT EC_50_ = 82.30 mg L^−1^). In contrast, *C. elegans* showed no mortality, suggesting lower terrestrial toxicity. These findings suggest that MAT and LAC may pose risks to aquatic ecosystems through runoff or leaching, necessitating further studies on their degradation products, soil microbiota, and non-target terrestrial organisms. Comparative analyses with conventional herbicides highlight MAT and LAC as selective, lower-impact alternatives. Future research should focus on their effects on terrestrial organisms, the ecological safety of degradation products, and large-scale bioassays to ensure their sustainability in agriculture.

## 1. Introduction

Agriculture faces numerous challenges, with biotic threats such as weeds ranking among the most significant due to their intense competition for resources like water, light, and inorganic nutrients [1,2]. Parasitic plants, a distinct category of weeds, are particularly problematic as they exacerbate these challenges. These plants produce abundant seeds capable of passive dispersal and long-term dormancy, remaining in the soil until a suitable host is detected [3]. Among these, broomrapes (*Orobanche* and *Phelipanche* spp.) are especially concerning, as they parasitize the roots of essential crops, including legumes and plants from the *Solanaceae* and *Compositae* families [4,5,6]. Their infestations can lead to severe economic losses, necessitating the adoption of integrated management strategies to mitigate their impact [7].

Efforts to control parasitic weeds using chemical herbicides have faced substantial limitations. The rapid adaptation of parasitic species and the emergence of herbicide resistance, often driven by the repeated use of herbicides with similar modes of action, have undermined their long-term effectiveness [8,9]. Moreover, the extensive application of herbicides poses environmental risks, including the contamination of soil, surface water, and groundwater, as well as impacts on non-target species [10]. Recent reviews have highlighted the toxicological effects of herbicides on non-target species and potential human health risks through exposure, thus emphasizing the need for safer alternatives [11].

These challenges have increased interest in natural products as alternatives to conventional herbicides. Many bioactive compounds derived from natural sources have shown promise in reducing seed banks or disrupting the early developmental stages of parasitic weeds [12]. For instance, a wide range of structurally diverse organic compounds have demonstrated herbicidal activity, either by inhibiting or stimulating the germination of parasitic weed seeds. These compounds include both naturally occurring substances and their derivatives and structural analogs [13,14,15,16]. One significant advantage of natural products lies in their potential to target novel pathways or mechanisms of action, offering solutions to herbicide resistance [17].

**Figure 1:**
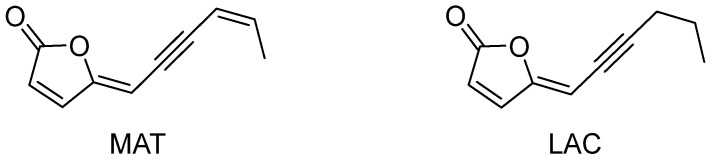
Structures of (4*Z*,8*Z*)-matricaria lactone (MAT) and (4*Z*)-lachnophyllum lactone (LAC).

Natural products are particularly well-suited for this purpose due to their evolutionary roles in plant defense, exemplified by allelochemicals, which function as natural herbicides [18]. However, sustainable agricultural practices demand that such compounds not only be effective but also exhibit minimal environmental impact. This study investigates the natural compounds (4*Z*,8*Z*)-matricaria lactone (MAT) and (4*Z*)-lachnophyllum lactone (LAC) (Figure 1), focusing on their stability and acute toxicity, thus obtaining insights into their potential applicability in bioherbicide formulations.

MAT and LAC are acetylenic furanones with herbicidal potential, particularly for controlling parasitic weeds. Their structures consist of a 2-furanone ring attached to an unsaturated chain, with MAT featuring an additional double bond at the C-8 in the Z configuration [19]. The selection of MAT and LAC as bioherbicide candidates is based on their bioactivity, lack of prior ecotoxicological studies, and structural simplicity compared to other bioactive compounds like strigolactones and sesquiterpene lactones. Their simpler structure may facilitate faster natural degradation, reducing ecological impact. Key structural features, such as the furanone ring and unsaturated exocyclic chain, suggest susceptibility to both abiotic and biotic degradation [20]. For example, alkaline soils may promote lactone ring opening via nucleophilic addition, while sunlight exposure could induce photolytic degradation. Additionally, microbial activity may contribute to enzymatic hydrolysis, and the system of conjugated bonds increases the likelihood of degradation through isomerization, hydrolysis, or polymerization.

MAT has been isolated from various *Asteraceae* species, including *Conyza* [21,22,23,24,25], *Erigeron* [26,27,28], and *Solidago* [29]. It exhibits strong inhibitory activity on the radicle growth of parasitic broomrape species [30], perennial plants [25], and phytopathogenic fungi [25,26,27]. LAC, isolated from *Conyza canadensis*, has demonstrated phytotoxicity against *Lactuca sativa*, *Agrostis stolonifera*, and *Lemna paucicostata* [25], as well as antifungal activity against *Colletotrichum* species affecting strawberries and *Penicillium digitatum*, the causative agent of citrus green mold [31]. Additionally, LAC strongly inhibits the growth of parasitic weeds, including *Orobanche crenata*, *O. cumana*, *O. minor*, and *Phelipanche ramosa* [30], and *Cuscuta campestris* [32]. Advances in the total synthesis of MAT and LAC have enabled their comprehensive evaluation for phytotoxicity against parasitic weeds (*O. minor*, *P. ramosa*, and *C. campestris*) and antifungal activity against *Verticillium dahliae* [33].

This study further evaluates their acute ecotoxicological profiles using representative aquatic organisms—*Aliivibrio fischeri* (bacterium), *Raphidocelis subcapitata* (alga), and *Daphnia magna* (crustacean)—as a baseline approach for non-target environmental impact. Additionally, *Caenorhabditis elegans* (nematode) and *Lepidum sativum* (plant) were tested, as they are widely used model organisms in ecotoxicology and plant sciences. *C. elegans* represents a non-target soil invertebrate, while *L. sativum*, a crop species, serves to assess potential unintended phytotoxic effects. Results from these tests, alongside the assessments of environmental stability, contribute to evaluating the feasibility of MAT and LAC for bioherbicide applications.

## 2. Results

### 2.1. Stability of MAT and LAC

The stability of MAT and LAC was first evaluated by direct phase thin-layer chromatography (TLC) for qualitative analysis. The chromatographic profiles of MAT at various exposure times revealed the presence of a brown spot at the baseline of the TLC, likely corresponding to degradation products formed during exposure. A similar spot was observed for LAC, though smaller in size. To quantify the degradation of both compounds, a quantitative analysis by HPLC was performed using calibration curves constructed from standard samples of MAT and LAC. These curves were prepared using solutions dissolved in CH_3_CN, with concentration ranges of 0.022–2.2 μg for MAT and 0.004–2.2 μg for LAC. The retention times were highly consistent, showing variations of less than 0.2 min, and the calibration curve characteristics are detailed in the Supplementary Information (Table S1).

This quantitative analysis revealed that both MAT and LAC degrade in ISO 8692:2012 medium under controlled temperature (20 °C) and light (254 nm) conditions over 16 days. At 20 °C, MAT degraded rapidly, with a degradation percentage of 89.74% after 1 day, increasing to 98.07% after 16 days. In contrast, LAC exhibited slower degradation, with 8.50% degradation after 1 day and 66.84% after 16 days. These results are summarized in Table 1.

### 2.2. Acute Toxicity of MAT and LAC

#### 2.2.1. Light Inhibition of *A. fischeri*

**Figure 2:**
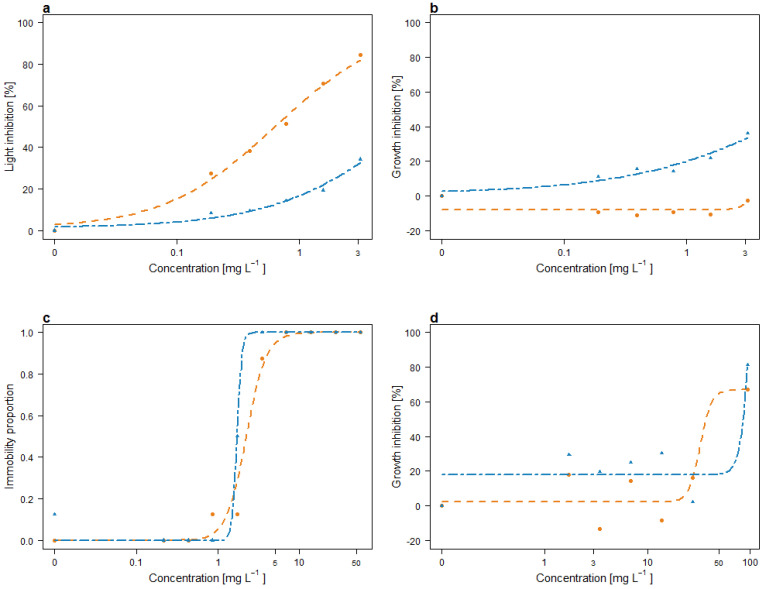
Concentration–response curves of aquatic species: *A. fischeri* (**a**), *R. subcapitata* (**b**), *D. magna* (**c**), and *L. sativum* (**d**) exposed to MAT (orange line) and LAC (blue line).

The light inhibition of *A. fischeri* after 30 min of exposure to varying concentrations of MAT and LAC is shown in Figure 2. MAT induced a stronger inhibitory effect compared to LAC, reaching approximately 80% inhibition at the highest tested concentration (3.125 mg L^−1^). In contrast, LAC caused less than 40% inhibition at the same concentration. The EC_10_ values, representing the concentrations causing 10% light inhibition, differing by an order of magnitude: 0.063 mg L^−1^ for MAT and 0.524 mg L^−1^ for LAC. For the EC_50_ (50% inhibition), a value of 0.642 mg L^−1^ was determined for MAT, while the EC_50_ for LAC could not be estimated within the tested concentration range, corresponding to 8.078 mg L^−1^.

#### 2.2.2. Growth Inhibition of *R. subcapitata*

The growth responses of *R. subcapitata* exposed to multiple concentrations of MAT and LAC are depicted in Figure 2. MAT exhibited a stimulatory effect on growth across all tested concentrations, with inhibition averaging approximately −10% at concentrations ranging from 0.19 to 1.56 mg L^−1^ and decreasing to −2.4% at the highest concentration (3.125 mg L^−1^). In contrast, LAC caused concentration-dependent inhibition, reaching a maximum of 36% at 3.125 mg L^−1^. Effective concentration values for MAT could not be estimated due to its stimulatory effects, whereas an EC_10_ of 0.304 mg L^−1^ was determined for LAC.

#### 2.2.3. Immobility of *D. magna*

The immobilization of *D. magna* after exposure to MAT and LAC is presented in Figure 2. MAT induced slightly higher immobilization compared to LAC. Both compounds caused complete immobilization (100%) at a concentration of 6.91 mg L^−1^ after 24 h of exposure. Immobilization increased with both concentration and exposure duration, leading to lower effective concentrations after 48 h. The EC_10_ and EC_50_ values after 48 h were 1.215 mg L^−1^ and 2.239 mg L^−1^ for MAT and 1.499 mg L^−1^ and 1.728 mg L^−1^ for LAC, respectively.

#### 2.2.4. Mortality of *C. elegans*

No mortality was observed in *C. elegans* after exposure to MAT and LAC across the tested concentrations after 24 h of exposure. This suggests that neither compound induces acute lethal effects in this model organism under the tested conditions.

#### 2.2.5. Germination and Growth of *L. sativum*

While *L. sativum* germination remained unaffected by MAT and LAC, both compounds caused a concentration-dependent reduction in shoot length, with growth inhibition increasing at higher concentrations. The EC_10_ and EC_50_ values for MAT were determined to be 38.48 mg L^−1^ and 82.30 mg L^−1^, respectively, while for LAC, they were estimated at 70.87 mg L^−1^ and 85.89 mg L^−1^.

The modeled curves used to estimate effective concentrations are presented in Figure 2. Table 2 summarizes the EC_10_ and EC_50_ values obtained for MAT and LAC across the tested species, along with their hazard classification scores. Based on the hazard classification, both compounds fall into Class II (slight acute hazard). The individual weight scores ranged from 0 to 3 for MAT and from 1 to 3 for LAC, with both compounds inducing 100% toxicity in *Daphnia magna*. Regarding the overall weight scores, both compounds yielded a value of 33.33%

The EC_50_ value for the positive control, potassium dichromate, was determined to be 3.730 mg L^−1^ in *A. fischeri*, 0.987 mg L^−1^ in *R. subcapitata*, and 10.501 mg L^−1^ and 5.66 mg L^−1^ in *D. magna* after 24 and 48 h, respectively. For the copper chloride, the EC_50_ value was determined to be 42.37 mg L^−1^ after 24 h of exposure. These values fall within the expected range reported in the literature, confirming the reliability and sensitivity of the assay [34,35,36,37,38].

### 2.3. Acute Toxicity of Commonly Used Herbicides

The acute toxicity of commonly used pesticides such as glyphosate, imzazapyr, imazaquin, and metolachlor on non-target species tested in this study were compared in terms of the EC_50_ obtained. The hazard ranking is also given in Table 3.

## 3. Discussion

Degradation studies reveal significant differences in the environmental stability of MAT and LAC, with implications for their persistence and ecological risks. MAT exhibited rapid degradation, decomposing by nearly 90% within 24 h and over 98% by day 16, likely due to its chemical structure and susceptibility to photolytic and hydrolytic processes. These results suggest that MAT applications could reduce environmental persistence, preventing long-term accumulation and minimizing lasting ecological impacts if degradation by-products are shown to be environmentally safe. In contrast, LAC degraded more slowly, with only 8.5% decomposed after one day and approximately 67% by day 16. This slower degradation makes LAC a potentially more effective allelochemical in contexts where long-lasting actions are needed. From a structural point of view, the faster degradation of MAT can be attributed to the additional double bond in the exocyclic chain, which is part of a conjugated system. This conjugated unsaturation in MAT likely increases its reactivity, making it more susceptible to photolytic and hydrolytic degradation compared to LAC.

These findings suggest that the differences in degradation rates between MAT and LAC could influence the compounds’ potential toxicity in different exposure scenarios. For instance, MAT’s rapid breakdown minimizes its long-term presence in the environment but may pose heightened risks during acute exposure events. Conversely, LAC’s moderate degradation rate increases the likelihood of prolonged presence in the environment, which could result in cumulative impacts on sensitive organisms over time.

Exposure to MAT resulted in a marked contrast in toxicity across test species. While *D. magna* and *A. fischeri* exhibited heightened sensitivity, with significant reductions in survival and bioluminescence, respectively, *L. sativum* and *R. subcapitata* displayed an unexpected increase in growth (a negative inhibition) at the concentrations tested. This differential response may be attributed to species-specific metabolic pathways and adaptive mechanisms. In aquatic organisms such as *Daphnia* and *Aliivibrio*, MAT may interfere with essential physiological processes, leading to acute toxicity. In contrast, in primary producers, low-dose hormetic effects or altered metabolic signaling may stimulate growth. Further investigations into the mode of action of MAT in plants and algae could help clarify whether this effect is due to hormesis, metabolic shifts, or interactions with plant growth regulators. In contrast, LAC exhibited a slower degradation rate and required higher concentrations to induce toxic effects. This suggests that its environmental persistence may contribute to prolonged but less immediate biological interactions. The need for higher concentrations to elicit toxicity indicates lower bioavailability or a different mode of action compared to MAT. While MAT showed rapid and pronounced effects on aquatic organisms, LAC’s delayed impact may be linked to its chemical stability, slower uptake, or reduced reactivity with biological targets.

The findings in *C. elegans* and *L. sativum* suggest that these compounds exhibit limited toxicity to soil-dwelling organisms and non-target terrestrial plants at the tested concentrations. The lack of significant mortality in *C. elegans* indicates that neither MAT nor LAC disrupt survival in this model species, while the unaffected germination of *L. sativum* suggests minimal phytotoxicity under these conditions. Notably, the concentrations required for effectiveness were an order of magnitude higher than those impacting aquatic species, reinforcing the selectivity of these compounds toward target weeds. Nevertheless, for practical field applications, it is essential that these compounds act at sufficiently low concentrations to minimize unintended effects and degrade rapidly to prevent runoff into aquatic ecosystems.

The effective concentrations studied against broomrape were found to be 100 mg L^−1^ for species like *O. minor*, *P. ramosa*, and *O. cumana* and 0.16 mg L^−1^ for *O. crenata* [30]. These concentrations are higher than those causing effects on aquatic species in the current study. While the low effects at similar concentrations in the two organisms tested suggest some degree of selectivity, the toxicity observed in aquatic organisms at lower concentrations raises concerns about potential environmental risks. These findings highlight the need for further studies to evaluate the suitability of MAT and LAC for practical use in weed management, taking into consideration both their efficacy and potential ecological impacts. Additionally, they highlight the importance of investigating the unknown risks associated with these compounds as bioproducts. The formation of degradation products, as observed through TLC, further emphasizes the necessity of evaluating their ecological impacts. While degradation generally reduces the toxicity of parent compounds, it can also result in by-products with distinct or even enhanced toxicological profiles, warranting closer examination.

In comparison, the toxicity of widely used herbicides like glyphosate, metolachlor, propanil, and atrazine varies significantly due to factors such as surfactants and commercial formulations [39,40,41,42,43,44,45,46,47,48,49,50,51,52,53]. Glyphosate, despite being controversial, remains the most widely used herbicide globally and is still approved for use in the EU until 2033 [54]. It has been classified as slightly to moderately toxic to amphibians, mammals, and aquatic invertebrates [55]. Atrazine has been identified as an endocrine disruptor [56], while metolachlor is considered a possible human carcinogen [57].

The comparison with existing herbicides highlights the potential benefits of MAT and LAC as selective alternatives. Future research should prioritize characterizing degradation products to assess their ecological safety and explore the effects of varying environmental conditions, such as temperature and pH, as well as the chronic exposure to multiple species to understand their behavior in more complex and realistic scenarios. Additionally, the challenges related to the scalability of production and suitable formulation need to be addressed for large-scale agricultural applications. MAT’s and LAC’s relatively simple structure could facilitate their synthesis through scalable synthetic methods, making them more accessible and cost-effective compared to other bioactive natural products.

## 4. Conclusions

MAT and LAC demonstrate potential as environmentally safer alternatives to conventional herbicides, particularly regarding their impact on model aquatic organisms, terrestrial species, and degradation profiles. MAT’s rapid degradation, coupled with its selective toxicity, minimizes the risks of long-term environmental contamination, while LAC, being more stable, exhibits lower toxicity in both the tested model organisms and terrestrial species like *C. elegans* and *L. sativum*. These findings suggest that both compounds could serve as promising candidates for further investigation regarding parasitic weed management. However, a comprehensive understanding of the ecological implications of their degradation products, their modes of action, and their behavior in field conditions is crucial for their successful integration into sustainable agricultural practices. While this study provides valuable insights into the acute effects on both aquatic and terrestrial organisms, additional research is necessary to assess the broader environmental impacts, particularly on terrestrial ecosystems, as well as to evaluate chronic and sub-lethal effects in various species.

## 5. Materials and Methods

### 5.1. General Experimental Procedures

The ^1^H NMR spectra were recorded at 500 MHz, in CDCl_3_ on a Varian spectrometer, and the same solvent was used as the internal standard. ESI mass spectra were obtained using the LC/MS TOF system AGILENT 6230B, HPLC 1260 Infinity. Column chromatography (CC) was performed using silica gel (Merck, Kieselgel 60, 0.063–0.200 mm). Analytical and preparative TLC was carried out on silica gel plates (Merck, Kieselgel 60, F_254_, 0.25 and 0.5 mm, respectively); the spots were visualized by exposure to UV light (254 nm) and/or iodine vapors and/or by first spraying with 10% H_2_SO_4_ in MeOH, and then with 5% phosphomolybdic acid in EtOH, followed by heating at 110 °C for 10 min. The HPLC system (HITACHI) consisted of a pump (5160) and a spectrophotometric detector (5410). The HPLC separations were performed using a Merck (Darmstadt, Germany) C_18_ reversed-phase column Lichrocart (250 × 4.6 mm i.d.; 5 μm).

### 5.2. Isolation and Identification of MAT and LAC

The MAT and LAC compounds were isolated following a procedure previously reported for the n-hexane extract of Conyza bonariensis shoots [30]. A total of 400 g of shoots were blended and extracted using H_2_O/MeOH (1/1, *v*/*v*; 1.5 L) under stirring conditions at room temperature for 24 h. The hydroalcoholic suspensions were then centrifuged (7000 rpm; 10 min) and extracted with n-hexane (3 × 0.5 L). The extract was dried over anhydrous Na_2_SO_4_ and the solvent evaporated under reduced pressure. This extraction procedure was performed twice, and 221.4 mg of n-hexane extract was obtained. The n-hexane extract was purified by column chromatography on Si-gel, eluted with CHCl_3_/i-propanol (9/1, *v*/*v*), yielding six homogeneous fractions (F1–F6). The residue of F2 (106.1 mg) was purified by preparative TLC eluted with n-hexane/EtOAc (75/25, *v*/*v*), yielding 41.5 mg of LAC and 16.0 mg of MAT. Their ^1^H NMR spectra (Figures S1 and S2) were in agreement with data previously reported for MAT [25] and for LAC [32].

### 5.3. Stability Studies

For the photodegradation assays, 3 mg of MAT and 3 mg of LAC were separately dissolved in 150 µL of DMSO and added to 3 mL of International Organization for Standardization (ISO) 8692:2012 medium. The solutions were exposed to 254 nm of light for up to 16 days. At specific time intervals (1, 2, 3, 6, and 16 days), 400 µL of the solutions were collected and analyzed by TLC using n-hexane/EtOAc (75:25, *v*/*v*) as the eluent, with MAT and LAC standard samples serving as references.

HPLC analysis was performed in isocratic mode and with the same ISO medium solutions, using MeCN–H_2_O (0.1% *v*/*v* formic acid) at a 60:40 (*v*/*v*) ratio as the mobile phase, with a flow rate of 0.5 mL/min. Detection was carried out at 220 nm with a 20 μL injection loop, monitoring the chromatogram for over 50 min to identify potential degradation products. Each sample was analyzed in triplicate. Control solutions containing only the culture medium and DMSO (without MAT or LAC) were also analyzed under identical conditions to ensure specificity.

Calibration curves for MAT and LAC were prepared by injecting standard samples at concentrations ranging from 0.022 to 2.2 mg for MAT and 0.004 to 2.2 mg for LAC. These standards were used to optimize the method and quantify the degradation products.

### 5.4. Ecotoxicity Analysis

The ISO 8692:2012 [58] medium was employed for the preparation of test solutions and for the control medium of the aquatic organisms. A stock solution with the concentration of 100 mg L^−^^1^ was used to prepare the concentrations tested for each organism.

#### 5.4.1. Luminescence Bacteria Inhibition

The bioluminescence inhibition test (30 min) was detected with the A. fischeri (NRRLB-11177) supplied by MicroBioTest, Gent, Belgium, and according to ISO 11348-3 [59]. The bioluminescence was determined by the luminometer Microtox (Model 500 analyzer, New Castle, DE, USA) at 490 nm. To provide the required osmotic pressure for the bacterium, the stock solution was diluted to perform the test in saline water solution (2% sodium chloride, NaCl). The concentrations tested correspond to 0.19, 0.39, 0.78, 1.56, and 3.12 mg L^−^^1^ and a negative control of 0 mg L^−^^1^. Toxicity tests were performed in triplicate with a control, and the percentage luminescence inhibition was expressed as the ratio of the decrease in bacterial light production to the remaining light.

#### 5.4.2. Algal Growth Inhibition

The algal growth inhibition test (72 h) with *R. subcapitata* was carried out according to ISO 8692 [58]. The algal density was determined by spectrophotometric analysis (DR5000, Hach Lange GbH, Weinheim, Germany) at 670 nm. The concentrations tested correspond to 0.19, 0.39, 0.78, 1.56, and 3.12 mg L^−^^1^ and a negative control of 0 mg L^−^^1^. The growth inhibition was calculated as the difference in growth at the end of the test of the sample and the control group. Toxicity tests were carried out in triplicate.

#### 5.4.3. Crustacean Immobility

The immobility acute test (48 h) with *D. magna* was conducted according to ISO 6341 [60]. *D. magna* was selected from laboratory stock cultures at the Hygiene Laboratory of the Department of Biology of the University of Naples Federico II, placed in ISO medium, and fed daily with the microalgae *R. subcapitata*. For the test, five neonates (third brood, <24 h old) were exposed in 10 mL of ISO medium to the following corresponding concentrations: 0.22, 0.43, 0.86, 1.73, 3.46, 6.91, 13.83, 27.65, and 55.30 mg L^−^^1^ and a negative control of 0 mg L^−^^1^. Each concentration had 4 replicates, and immobility was assessed after 24 and 48 h of exposure.

#### 5.4.4. Nematodes Survival

The nematode bioassay was carried out using the Caenorhabditis elegans wild-type strain N2, Bristol variant, according to the ASTM E2172—01 Standard Method (2014) [61]. Culture was maintained on NGM (nematode growth media) plates seeded with *Escherichia coli* (uracil-deficient OP50 strain) and stored at 20 °C. The tests were conducted using age-synchronized adult nematodes, obtained by lysing gravid nematodes with a bleaching solution (10 g/L NaOH, 10.5 g/L NaOCl), followed by centrifugation and washing with an M9 mineral medium (2.2 mM KH_2_PO_4_, 4.2 mM Na_2_HPO_4_, 85.6 mM NaCl, and 1 mM MgSO_4_). The synchronized nematodes were then transferred to NGM agar plates and left to recover overnight.

Soil samples (2.33 g) were hydrated to 35–45% of their dry weight using K-medium in centrifuge tubes. Ten worms were transferred to each tube and exposed to the soil samples for 24 h at 20 °C. The exposure concentrations were 1.73, 3.46, 6.91, 13.83, 27.65, and 94 mg L^−^^1^ and a negative control of 0 mg L^−^^1^. Treatments were performed in triplicate, with worms kept unfed during the exposure period. Nematodes were extracted by centrifugation (2000 rpm, 2 min) using silica gel (Ludox TM 50, Sigma-Aldrich, St. Louis, MO, USA) with a specific density of 1.13 g/mL. The supernatant was then transferred to 100 mm glass Petri dishes and washed with Keller (K) medium. Extracted individuals were counted under a microscope (40× magnification), with mortality as the measured endpoint. Test validity required a minimum of 80% nematode recovery and 90% survival in the control group.

#### 5.4.5. Plant Growth and Germination

*Lepidum sativum* seeds were purchased from Microbiotests (Gent, Belgium) and were used for the phytotoxicity test. Tests were conducted following the standard method [62]. Seeds (*n* = 10) were placed in 10 g of soil medium and exposure was carried out in triplicates. The exposure concentrations were 1.73, 3.46, 6.91, 13.83, 27.65, and 94 mg L^−^^1^ and a negative control of 0 mg L^−^^1^. Test organisms were incubated at 25 ± 1 °C in darkness and the number of seeds germinated and the length of the developing roots were measured after 3 days. Germination (%) and root elongation inhibition were combined to calculate the germination index (GI, %).

### 5.5. Data Analysis

Data analysis was conducted using RStudio for Windows (Version 2024.09.1) [63]. Dose–response models were applied to estimate the effective concentrations (EC_10_ and EC_50_) for the tested endpoints using the “drc” package [64]. For *D. magna*, the data were modeled with a binomial log-logistic approach, whereas data for *R. subcapitata*, *A. fischeri* and *L. sativum* were fitted to log-logistic models with the upper limit constrained to 100. Furthermore, to ensure assay sensitivity and validity, a positive control exposed to potassium dichromate for the aquatic species and copper chloride for *C. elegans* was tested under the same experimental conditions. EC_50_ values were determined for the positive control to confirm the assay’s reliability.

When possible, the EC_50_ values derived from the tests were used for toxicity classification following the methodology in [65]. Compounds were categorized into five hazard classes, ranging from Class I (no acute hazard) to Class V (very high acute hazard). Additionally, weight scores for each test were calculated to quantify the relative level of toxicity, with scores assigned as follows: 0 (no significant toxic effect), 1 (significant toxic effect), 2 (toxic effect), and 3 (100% toxic effect). The weight score for each compound, based on the tests performed, was expressed as a percentage. For *D. magna*, hazard classification was based exclusively on the 48 h EC_50_ values, excluding those estimated at 24 h.

Furthermore, the EC_50_ values were compared to published data, and hazard rankings were assigned according to the Globally Harmonized System of Classification and Labeling of Chemicals (GHS) [66]. The classification criteria were as follows: very toxic (≤1 mg L^−1^), toxic (>1 mg L^−1^ and ≤10 mg L^−1^), harmful (>10 mg L^−1^ and ≤100 mg L^−1^), and not harmful (>100 mg L^−1^).

## Figures and Tables

**Table 1:** The amount of MAT and LAC detected at different exposition times and the percentages of the degraded compounds.

Compound	Exposition Time (Day)	Degraded Compound (%)
LAC	1	8.50
	2	19.23
	3	26.58
	6	28.24
	16	66.84
MAT	1	89.74
	2	90.06
	3	90.38
	6	94.55
	16	98.07

**Table 2:** EC_10_ and EC_50_ values for MAT and LAC after exposure of *A. fischeri*, *R. subcapitata*, and *D. magna*; values are in mgL^−1^; EC = effective concentration; ±95% standard error in brackets (*n* = 3).

Compound	Organism	Exposure Time	EC_10_ [mg L^−1^]	EC_50_ [mg L^−1^]	Test Score	Weight Score [%]
MAT	*A. fischeri*	30 min	0.063 (0.013)	0.642 (0.052)	2	
	*R. subcapitata*	72 h	3.417 (0.620) *	4.52 (3.113) *	0	46.66
	*D. magna*	24 h	3.11 (1.894)	3.56 (0.673)	NC	
		48 h	1.21 (0.294)	2.23 (0.349)	3	
	*C. elegans*	24 h	NC	NC	0	
	*L. Sativum*	72 h	38.48 (23.979)	82.30 (15.421)	2	
LAC	*A. fischeri*	30 min	0.524 (0.212)	8.07 (2.052) *	1	
	*R. subcapitata*	72 h	0.304 (0.125)	9.88 (2.509) *	1	
	*D. magna*	24 h	1.67 (0.313)	2.44 (0.322)	NC	53.33
		48 h	1.49 (1.063)	1.72 (0.079)	3	
	*C. elegans*	24 h	NC	NC	0	
	*L. Sativum*	72 h	70.87 (195.32)	85.89 (75.69)	3	

* Values estimated from the model, outside of the tested concentrations.

**Table 3:** EC_50_ values from the literature for the aquatic test species exposed to multiple commonly used herbicides.

Chemical Group	Active Ingredient	Organism	Exposure Time	Endpoint	EC_50_ [mg L^−1^]	Hazard Ranking	Reference
Organophosphorus	Glyphosate	*D. magna*	48 h	EC_50_	7.15 °	***	[39]
			48 h	EC_50_	4.30 °°	***	[39]
			48 h	EC_50_	4.20	***	[40]
			48 h	EC_50_	8.90	***	[41]
			48 h	LC_50_	21.34	**	[42]
			48 h	LC_50_	190.0	*	[43]
			48 h	LC_50_	0.012	****	[44]
			48 h	LC_50_	9.34	***	[45]
			48 h	LC_50_	11.68	**	[46]
		*R. subcapitata*	96 h	EC_50_	5.55	***	[47]
		*A. fischeri*	30 min	EC_50_	2.93	***	[48]
Chloroacetamide	Metolachlor	*D. magna*	48 h	EC_50_	23.50	**	[49]
		*R. subcapitata*	96 h	EC_50_	5.51	***	[47]
			72 h	EC_50_	0.055	****	[49]
		*A. fischeri*	30 min	EC_50_	265.00	*	[50]
			30 min	EC_50_	17.00	**	[49]
			30 min	EC_50_	214.85	*	[51]
Anilide	Propanil	*D. magna*	48 h	EC_50_	2.00	***	[49]
		*R. subcapitata*	72 h	EC_50_	0.05	****	[49]
Triazine	Atrazine	*D. magna*	48 h	EC_50_	0.05	****	[49]
		*R. subcapitata*	72 h	EC_50_	0.02	****	[49]
		*A. fischeri*	30 min	EC_50_	39.80	**	[49]
	MAT	*A. fischeri*	30 min	EC_50_	0.642	****	This study
		*R. subcapitata*	72 h	EC_50_	4.520	***	
		*D. magna*	48 h	EC_50_	2.239	***	
	LAC	*A. fischeri*	30 min	EC_50_	8.078	***	
		*R. subcapitata*	72 h	EC_50_	9.880	***	
		*D. magna*	48 h	EC_50_	1.728	***	

**** Very toxic (≤1 mg L^−1^); *** toxic (<1 mg L^−1^ ≤ 10 mg L^−1^); ** harmful: (<10 mg L^−1^ ≤ 100 mg L^−1^); * not harmful: (>100 mg L^−1^). ° average of multiple tests performed, rounded up (formulation commercially available). °° average of multiple tests performed, given as isopropylamine salt.

## Data Availability

The original contributions presented in this study are included in the article/Supplementary Material. Further inquiries can be directed to the corresponding author(s).

## Supplementary Materials

The following supporting information can be downloaded at: https://www.mdpi.com/article/10.3390/toxins17040169/s1, Figure S1: ^1^H-NMR spectrum of (4*Z*,8*Z*)-matricaria lactone (MAT) recorded in CDCl_3_ at 500 MHz; Figure S2: ^1^H-NMR spectrum of (4*Z*)-lachnophyllum lactone (LAC) recorded in CDCl_3_ at 500 MHz; Table S1: Analytical characteristics of the calibration curves^1^ for the quantification of MAC and LAC by HPLC.

## References

[1] Oerke E.-C. (2006). Crop losses to pests. J. Agric. Sci..

[2] Chauhan B.S. (2020). Grand challenges in weed management. Front. Agron..

[3] Macias F.A., Garcia-Diaz M.D., Perez-de-Luque A., Rubiales D., Galindo J.C. (2009). New chemical clues for broomrape-sunflower host− parasite interactions: Synthesis of guaianestrigolactones. J. Agric. Food Chem..

[4] Dhanapal G., Struik P., Udayakumar M., Timmermans P. (1996). Management of broomrape (*Orobanche* spp.)—A review. J. Agron. Crop Sci..

[5] Cardoso C., Ruyter-Spira C., Bouwmeester H.J. (2011). Strigolactones and root infestation by plant-parasitic *Striga*, *Orobanche* and *Phelipanche* spp.. Plant Sci..

[6] Soto-Cruz F.J., Zorrilla J.G., Rial C., Varela R.M., Molinillo J.M., Igartuburu J.M., Macías F.A. (2021). Allelopathic activity of strigolactones on the germination of parasitic plants and arbuscular mycorrhizal fungi growth. Agronomy.

[7] Rubiales D., Fernández-Aparicio M. (2012). Innovations in parasitic weeds management in legume crops. A review. Agron. Sustain. Dev..

[8] Fernández-Aparicio M., Delavault P., Timko M.P. (2020). Management of infection by parasitic weeds: A review. Plants.

[9] Harrington K.C., Ghanizadeh H. (2024). Comparing herbicide resistance in New Zealand and Australia. N. Z. J. Agric. Res..

[10] Nath C.P., Singh R.G., Choudhary V.K., Datta D., Nandan R., Singh S.S. (2024). Challenges and alternatives of herbicide-based weed management. Agronomy.

[11] Bamal D., Duhan A., Pal A., Beniwal R.K., Kumawat P., Dhanda S., Goyat A., Hooda V.S., Yadav R. (2024). Herbicide risks to non-target species and the environment: A review. Environ. Chem. Lett..

[12] Sauerborn J., Müller-Stöver D., Hershenhorn J. (2007). The role of biological control in managing parasitic weeds. Crop Prot..

[13] Joel D.M., Chaudhuri S.K., Plakhine D., Ziadna H., Steffens J.C. (2011). Dehydrocostus lactone is exuded from sunflower roots and stimulates germination of the root parasite *Orobanche cumana*. Phytochemistry.

[14] Okazawa A., Noda S., Mimura Y., Fujino K., Wakabayashi T., Ohta D., Sugimoto Y., Sonoda M. (2023). The structure-activity relationship of aryloxyacetylthioureas for the inhibition of *Orobanche minor* radicle elongation. J. Pestic. Sci..

[15] Zorrilla J.G., Innangi M., Cala Peralta A., Soriano G., Russo M.T., Masi M., Fernández-Aparicio M., Cimmino A. (2024). Sesquiterpene Lactones Isolated from *Centaurea cineraria* L. subsp. *cineraria* Inhibit the Radicle Growth of Broomrape Weeds. Plants.

[16] Ge Y., Chen X., Khan S.N., Jia S., Chen G. (2023). Synthesis and germination activity study of novel strigolactam/strigolactone analogues. Tetrahedron Lett..

[17] Qu R.Y., He B., Yang J.F., Lin H.Y., Yang W.C., Wu Q.Y., Li Q.X., Yang G.F. (2021). Where are the new herbicides?. Pest Manag. Sci..

[18] Macías F.A., Molinillo J.M., Galindo J.C., Varela R.M., Simonet A.M., Castellano D. (2001). The use of allelopathic studies in the search for natural herbicides. J. Crop Prod..

[19] Soriano G., Siciliano A., Fernández-Aparicio M., Cala Peralta A., Masi M., Moreno-Robles A., Guida M., Cimmino A. (2022). Iridoid glycosides isolated from *Bellardia trixago* identified as inhibitors of *Orobanche cumana* radicle growth. Toxins.

[20] Mordi R.C. (1993). Mechanism of beta-carotene degradation. Biochem. J..

[21] Metwally M.A., Dawidar A.A.M. (1984). Constituents of *Conyza aegyptiaca* L.. Pharmazie.

[22] Hrutfiord B.F., Hatheway W.H., Smith D.B. (1988). Essential oil of *Conyza canadensis*. Phytochemistry.

[23] Barbosa L.C., Paula V.F., Azevedo A.S., Silva E.A., Nascimento E.A. (2005). Essential oil composition from some plant parts of *Conyza bonariensis* (L.) Cronquist. Flavour Fragr. J..

[24] Csupor-Löffler B., Hajdú Z., Zupkó I., Molnár J., Forgo P., Vasas A., Kele Z., Hohmann J. (2011). Antiproliferative constituents of the roots of *Conyza canadensis*. Planta Med..

[25] Queiroz S.C., Cantrell C.L., Duke S.O., Wedge D.E., Nandula V.K., Moraes R.M., Cerdeira A.L. (2012). Bioassay-directed isolation and identification of phytotoxic and fungitoxic acetylenes from *Conyza canadensis*. J. Agric. Food Chem..

[26] Sorensen J., Sorensen N. (1969). Studies related to naturally occurring acetylene compounds. XXXV. Investigation of *Erigeron* spp. from the Australian mountains and Tasmania. Aust. J. Chem..

[27] Vidari G., Abdo S., Gilardoni G., Ciapessoni A., Gusmeroli M., Zanoni G. (2006). Fungitoxic metabolites from *Erigeron apiculatus*. Fitoterapia.

[28] Nazaruk J., Kalemba D. (2009). Chemical composition of the essential oils from the roots of *Erigeron acris* L. and *Erigeron annuus* (L.) Pers. Molecules.

[29] Lam J. (1971). Polyacetylenes of *Solidago virgaurea*: Their seasonal variation and NMR long-range spin coupling constants. Phytochemistry.

[30] Peralta A.C., Soriano G., Zorrilla J.G., Masi M., Cimmino A., Fernández-Aparicio M. (2022). Characterization of *Conyza bonariensis* allelochemicals against broomrape weeds. Molecules.

[31] Terao D., Queiroz S.C.N., Maia A.D.H.N. (2022). Bioactive compound isolated from *Conyza canadensis* combined with physical treatments for the control of green mould in Orange. J. Phytopathol..

[32] Fernández-Aparicio M., Soriano G., Masi M., Carretero P., Vilariño-Rodríguez S., Cimmino A. (2022). (4Z)-Lachnophyllum lactone, an acetylenic furanone from *Conyza bonariensis*, identified for the first time with allelopathic activity against *Cuscuta campestris*. Agriculture.

[33] Soriano G., Arnodo D., Masi M., Fernández-Aparicio M., Landa B.B., Olivares-García C., Cimmino A., Prandi C. (2024). (4Z)-Lachnophyllum Lactone, a Metabolite with Phytotoxic and Antifungal Activity against Pests Affecting Mediterranean Agriculture: A New Versatile and Easy Scalable Parallel Synthesis. J. Agric. Food Chem..

[34] Gopi R., Ayyappan S., Chandrasehar G., Krishna V., Goparaju A. (2012). Effect of potassium dichromate on the survival and reproduction of *Daphnia magna*. Bull. Environ. Pharmacol. Life Sci..

[35] Sudha V., Baskar K., Tamilselvan C. (2016). Immobilization effect of Potassium dichromate on *Daphnia magna* (Straus). Eur. J. Environ. Ecol..

[36] Kikuchi M., Syudo A., Hukumori M., Naito C., Sawai J. (2017). Changes in aquatic toxicity of potassium dichromate as a function of water quality parameters. Chemosphere.

[37] Santos M., Vicensotti J., Monteiro R.T.R. (2007). Sensitivity of four test organisms (*Chironomus xanthus*, *Daphnia magna*, *Hydra attenuata* and *Pseudokirchneriella subcapitata*) to NaCl: An alternative reference toxicant. J. Braz. Soc. Ecotoxicol..

[38] Wimmerova L., Solcova O., Spacilova M., Cehajic N., Krejcikova S., Marsik P. (2022). Toxicity Assessment and Treatment Options of Diclofenac and Triclosan Dissolved in Water. Toxics.

[39] Cuhra M., Traavik T., Bøhn T. (2013). Clone-and age-dependent toxicity of a glyphosate commercial formulation and its active ingredient in *Daphnia magna*. Ecotoxicology.

[40] Sihtmäe M., Blinova I., Künnis-Beres K., Kanarbik L., Heinlaan M., Kahru A. (2013). Ecotoxicological effects of different glyphosate formulations. Appl. Soil Ecol..

[41] Hansen L.R., Roslev P. (2016). Behavioral responses of juvenile *Daphnia magna* after exposure to glyphosate and glyphosate-copper complexes. Aquat. Toxicol..

[42] Gustinasari K., Sługocki Ł., Czerniawski R., Pandebesie E.S., Hermana J. (2021). Acute toxicity and morphology alterations of glyphosate-based herbicides to *Daphnia magna* and *Cyclops vicinus*. Toxicol. Res..

[43] Raipulis J., Toma M., Balode M. (2009). Toxicity and genotoxicity testing of roundup. Proc. Latv. Acad. Sci. Sect. B Nat. Exact Appl. Sci..

[44] Sarigül Z., Bekcan S. (2009). Acute toxicity of the herbicide glyphosate on *Daphnia magna*. J. Agric. Sci..

[45] Demetrio P.M., Bonetto C., Ronco A.E. (2014). The effect of cypermethrin, chlorpyrifos, and glyphosate active ingredients and formulations on *Daphnia magna* (Straus). Bull. Environ. Contam. Toxicol..

[46] Reno U., Doyle S.R., Momo F.R., Regaldo L., Gagneten A.M. (2018). Effects of glyphosate formulations on the population dynamics of two freshwater cladoceran species. Ecotoxicology.

[47] Ma J., Wang S., Wang P., Ma L., Chen X., Xu R. (2006). Toxicity assessment of 40 herbicides to the green alga *Raphidocelis subcapitata*. Ecotoxicol. Environ. Saf..

[48] Vurm R., Tajnaiová L., Kofroňová J. (2021). The Influence of Herbicides to Marine Organisms *Aliivibrio fischeri* and *Artemia salina*. Toxics.

[49] Köck M., Farré M., Martínez E., Gajda-Schrantz K., Ginebreda A., Navarro A., de Alda M.L., Barceló D. (2010). Integrated ecotoxicological and chemical approach for the assessment of pesticide pollution in the Ebro River delta (Spain). J. Hydrol..

[50] Tóth G., Háhn J., Kriszt B., Szoboszlay S. (2019). Acute and chronic toxicity of herbicides and their mixtures measured by *Aliivibrio fischeri* ecotoxicological assay. Ecotoxicol. Environ. Saf..

[51] Osano O., Admiraal W., Klamer H.J.C., Pastor D., Bleeker E.A.J. (2002). Comparative toxic and genotoxic effects of chloroacetanilides, formamidines and their degradation products on *Vibrio fischeri* and *Chironomus riparius*. Environ. Pollut..

[52] Liu Z., Zhang P., Yang J., Gao Y., Fan J., Fan R. (2021). Effects of Imidacloprid Applied Alone or in Combination with Organosilicone Surfactants on Biological Traits and Predatory Feeding of *Chrysoperla nipponensis* (Neuroptera: Chrysopidae). J. Econ. Entomol..

[53] Annett R., Habibi H.R., Hontela A. (2014). Impact of glyphosate and glyphosate-based herbicides on the freshwater environment. J. Appl. Toxicol..

[54] Authority E.F.S., Agency E.C. (2024). Technical and scientific assistance on the internal review under Regulation (EC) No 1367/2006 of Commission Implementing Regulation (EU) 2023/2660 renewing the approval of the active substance glyphosate in accordance with Regulation (EC) No 1107/2009. EFSA Support. Publ..

[55] Mesnage R., Defarge N., Spiroux de Vendômois J., Séralini G.E. (2015). Potential toxic effects of glyphosate and its commercial formulations below regulatory limits. Food Chem. Toxicol..

[56] Gupta P.K., Gupta R.C. (2018). Chapter 44—Toxicity of Herbicides. Veterinary Toxicology.

[57] Heydens W.F., Lamb I.C., Wilson A.G.E., Krieger R. (2010). Chapter 82—Chloracetanilides. Hayes’ Handbook of Pesticide Toxicology.

[58] (2012). Water Quality—Fresh Water Algal Growth Inhibition Test with Unicellular Green Algae.

[59] (2007). Water Quality—Determination of the Inhibitory Effect of Water Samples on the Light Emission of *Vibrio fischeri* (Luminescent Bacteria Test)—Part 3: Method Using Freeze-Dried Bacteria.

[60] (2012). Water Quality—Determination of the Inhibition of the Mobility of *Daphnia magna* Straus (Cladocera, Crustacea)—Acute Toxicity Test.

[61] (2022). Standard Guide for Conducting Laboratory Soil Toxicity Tests with the Nematode *Caenorhabditis elegans*.

[62] (2013). Soil Quality—Assessment of Genotoxic Effects on Higher Plants—*Vicia faba* Micronucleus Test.

[63] Team P. (2024). RStudio: Integrated Development Environment for R [Computer Software].

[64] Ritz C., Baty F., Streibig J.C., Gerhard D. (2015). Dose-response analysis using R. PLoS ONE.

[65] Persoone G., Marsalek B., Blinova I., Törökne A., Zarina D., Manusadzianas L., Nalecz-Jawecki G., Tofan L., Stepanova N., Tothova L. (2003). A practical and user-friendly toxicity classification system with microbiotests for natural waters and wastewaters. Environ. Toxicol. Int. J..

[66] Secretariat U.E. (2023). Globally Harmonized System of Classification and Labelling of Chemicals (GHS).

