# Supplementary material for: Toxicity Assessment of (4Z)-Lachnophyllum and (4Z,8Z)-Matricaria Lactones: Implications for Environmental Safety of Bioherbicides
Source: Toxins (Basel). 2025 Apr 1;17(4):169. doi: 10.3390/toxins17040169 (PMC12031237; doi:10.3390/toxins17040169)
Supplement: Supplementary file 1 [file toxins-17-00169-s001.zip › toxins-3445023-supplementary.docx]

**Supporting Information**

**Ecotoxicological Evaluation of (4*Z*)-Lachnophyllum Lactone and (4*Z*,8*Z*)-Matricaria Lactone as Potential Bioherbicides for Parasitic Weed Management**

**Supporting Information list**

**Figure S1.** ^1^H-NMR spectrum of (4*Z*,8*Z*)-matricaria lactone (MAT) recorded in CDCl_3_ at 500 MHz.

**Figure S2.** ^1^H-NMR spectrum of (4*Z*)-lachnophyllum lactone (LAC) recorded in CDCl_3_ at 500 MHz.

**Table S1.** Analytical characteristics of the calibration curves^1^ for the quantification of MAC and LAC by HPLC.


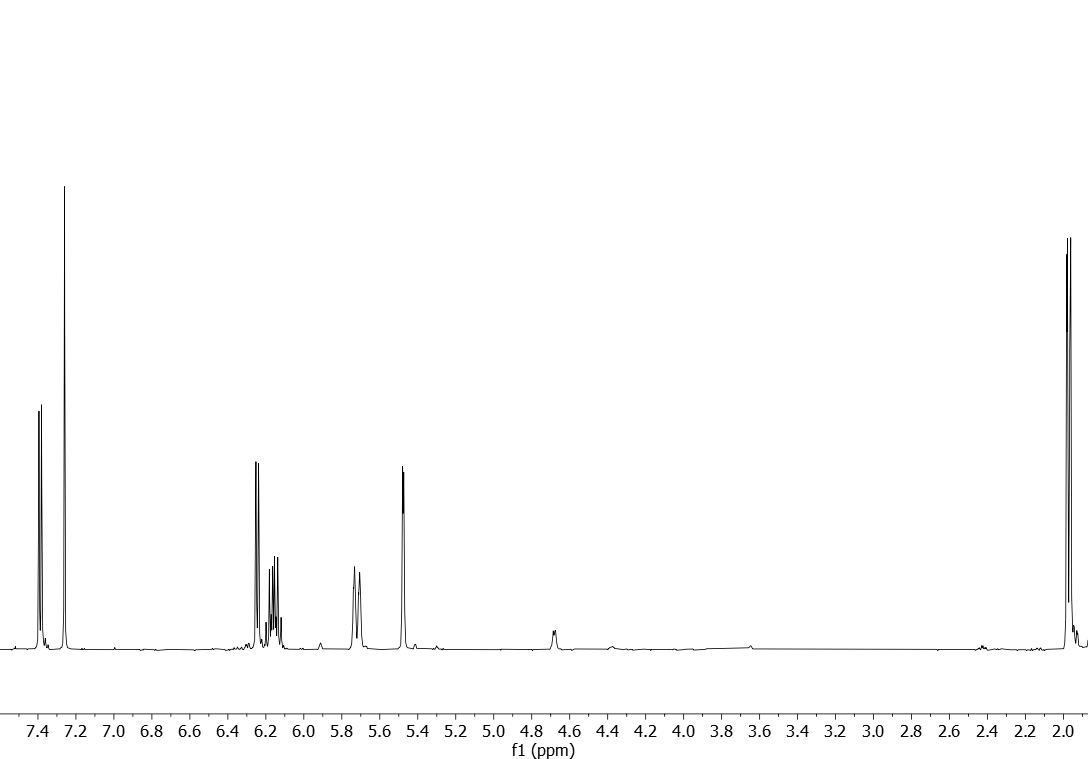


**Figure S1.** ^1^H-NMR spectrum of (4*Z*,8*Z*)-matricaria lactone (MAT) recorded in CDCl_3_ at 500 MHz.


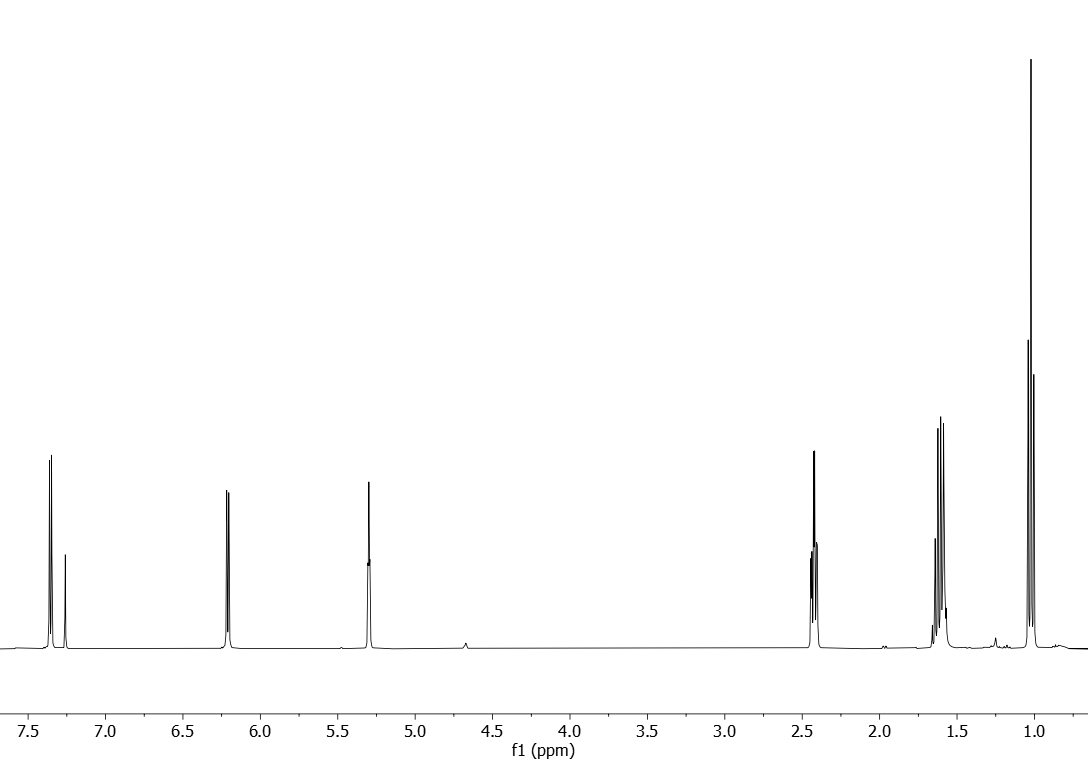


**Figure S2.** ^1^H-NMR spectrum of (4*Z*)-lachnophyllum lactone (LAC) recorded in CDCl_3_ at 500 MHz.

**Table S1.** Analytical characteristics of the calibration curves for the quantification of MAC and LAC by HPLC.^1^

| **Compound** | **Retention time (min)** | **R^2^** | **Data points** | **Limit of detection (µg)** |
| --- | --- | --- | --- | --- |
| MAC | 6.012 | 0.998 | 20 | 0.022 |
| LAC | 8.551 | 0.999 | 20 | 0.004 |

^1^ The calibration curves were calculated with the equation form “y = a + bx”, where the “y” is the peak area and “x” the amount (µg) of MAC and LAC injected.
